# Nonpharmacological Modulation of Chronic Inflammation in Parkinson's Disease: Role of Diet Interventions

**DOI:** 10.1155/2019/7535472

**Published:** 2019-08-25

**Authors:** Stefania Kalampokini, Anouck Becker, Klaus Fassbender, Epameinondas Lyros, Marcus M. Unger

**Affiliations:** Department of Neurology, University Hospital of Saarland, Kirrberger Straße, 66421 Homburg, Germany

## Abstract

Neuroinflammation is increasingly recognized as an important pathophysiological feature of neurodegenerative diseases such as Parkinson's disease (PD). Recent evidence suggests that neuroinflammation in PD might originate in the intestine and the bidirectional communication between the central and enteric nervous system, the so-called “gut-brain axis,” has received growing attention due to its contribution to the pathogenesis of neurological disorders. Diet targets mediators of inflammation with various mechanisms and combined with dopaminergic treatment can exert various beneficial effects in PD. Food-based therapies may favorably modulate gut microbiota composition and enhance the intestinal epithelial integrity or decrease the proinflammatory response by direct effects on immune cells. Diets rich in pre- and probiotics, polyunsaturated fatty acids, phenols including flavonoids, and vitamins, such as the Mediterranean diet or a plant-based diet, may attenuate chronic inflammation and positively influence PD symptoms and even progression of the disease. Dietary strategies should be encouraged in the context of a healthy lifestyle with physical activity, which also has neuroimmune-modifying properties. Thus, diet adaptation appears to be an effective additive, nonpharmacological therapeutic strategy that can attenuate the chronic inflammation implicated in PD, potentially slow down degeneration, and thereby modify the course of the disease. PD patients should be highly encouraged to adopt corresponding lifestyle modifications, in order to improve not only PD symptoms, but also general quality of life. Future research should focus on planning larger clinical trials with dietary interventions in PD in order to obtain hard evidence for the hypothesized beneficial effects.

## 1. Introduction

Parkinson's disease (PD) is a neurodegenerative disorder characterized by a loss of dopaminergic neurons in the substantia nigra (SN) as well as nondopaminergic neurons, including cholinergic neurons, norepinephrinergic neurons, serotoninergic neurons, and neurons of the enteric nervous system [1, 2]. The disease is also characterized by intracellular inclusions (so-called Lewy bodies) composed of fibrillar alpha-synuclein (a-Syn) and ubiquitinated proteins within neurons in various brain regions [3]. Despite the controversies with regard to the general validity of Braak's hypothesis and reasonable criticism to some aspects of his model, the gastrointestinal tract is widely considered as one possible ignition source of PD pathology [2, 4].

The assumed mechanisms of neurodegeneration in PD comprise oxidative stress, mitochondrial dysfunction, abnormal a-Syn oligomerization, and rise in iron content [5]. Furthermore, there is growing evidence suggesting that neuroinflammation is involved in the pathological process of PD [6–9]. Neuroinflammation plays a role in a variety of neurological disorders and has been implicated in other neurodegenerative disorders including Alzheimer's disease, Huntington's disease, amyotrophic lateral sclerosis, and progressive supranuclear palsy [10, 11]. Neuroinflammation involves microglial activation with production of cytokines and reactive oxygen species (ROS), astrogliosis, and lymphocytic infiltration [10]. Moreover, recent evidence suggests that chronic intestinal inflammation may contribute to the development of PD [12].

Dopamine replacement therapy, which remains the main pharmacological treatment in PD, alleviates the motor symptoms of the disease such as rigidity, resting tremor, and bradykinesia, while it influences to a lesser extent the nonmotor symptoms such as autonomic dysfunction, sensory, and neuropsychiatric disorders [13], has side effects, and has no proven effect on slowing down disease progression. For this reason, there is an urgent need to develop new additive therapeutic strategies targeting PD pathogenesis. There is growing evidence that diet [14, 15] can attenuate the neuroinflammation implicated in the pathophysiology of PD, rendering it an attractive nonpharmacological modulator of chronic inflammation in PD.

## 2. Chronic Inflammation in Parkinson's Disease

### 2.1. Neuroinflammation

Clinical and experimental evidence suggests that neuroinflammation may contribute to the progressive loss of dopaminergic neurons in PD. Data from postmortem studies showed the presence of activated microglial cells [16, 17], as well as a significant increase of glial cells expressing tumor necrosis factor-*α* (TNF-*α*), interleukin-1*β* (IL-1*β*), and interferon-gamma (IFN-*γ*) [18] within the SN of PD patients. Furthermore, the concentrations of TNF-*α*, IL-1*β*, IL-2, IL-4, IL-6, epidermal growth factor (EGF), transforming growth factor-a (TGF-a), TGF-*β*1, and *β*2-microglobulin were found to be increased in the striatum of PD patients [8]. CD4^+^ and CD8^+^ T cells were also found postmortem near dopaminergic neurons in the SN of both PD patients and 1-methyl-4-phenyl-1,2,3,6-tetrahydropyridine (MPTP) mouse models of PD [6]. Concentrations of several proinflammatory cytokines such as IL-1*β*, IL-2, IL-6, IL-10, and TNF-*α* were found to be elevated in the serum of PD patients [19, 20]. Furthermore, IL-1*β*, IL-6, TNF-*α*, and osteopontin were found to be elevated in the cerebrospinal fluid (CSF) of PD patients [8]. Associations have also been found between gene polymorphisms for TNF-*α* [9], IL-1*β* [21], as well as the histocompatibility human leucocyte antigen HLA-DRB5 gene [22], and increased risk of PD. While the exact effects induced by these polymorphisms are not clear, they might affect the basal level of the inflammatory status or the response to inflammatory stimuli [10]. In vivo imaging studies of microglia activation showed increased inflammation levels in the pons, striatum, and frontal, temporal, and occipital cortical regions in PD patients compared to age-matched healthy controls [7, 23]. Moreover, PET imaging studies showed that increased microglia activation correlated with reduced glucose metabolism and worse performance in general cognitive testing (Mini-Mental State Examination) in patients with Parkinson's disease dementia (PDD) [23, 24]. Epidemiological studies showed inconsistent data regarding the use of anti-inflammatory drugs (NSAID) and the risk of PD [25]. Nevertheless, ibuprofen users showed a significantly lower risk (RR 0.62) of developing PD compared to nonusers in a dose-dependent manner [26]. In MPTP animal models of PD, microglial activation is a common feature [27–29] and this seems to precede the degeneration of neurons [6, 28]. Furthermore, intranigral [30] or systemic injection of lipopolysaccharide (LPS) [31], which are part of the membrane of Gram-negative bacteria and can induce toxic and inflammatory effects by activating microglia, was found to cause death of dopaminergic neurons in animal models of PD. An astroglial [28] as well as lymphocytic infiltration of CD4^+^ and CD8^+^ T cells [6, 32] was also found in the SN and striatum of MPTP model animals.

The mechanisms of inflammation involve activation of microglia, accumulation of cytokines, nuclear factor kappa B (NF-*κ*B) pathway activation, and oxidative damage to proteins in the CSF and brain of PD patients [10]. Astroglial reaction, on the other hand, seems to play a role in neuroinflammation in PD, but it is unlikely to induce cell death as it takes place rather late in the degeneration process [10]. The immune cells of the systemic circulation may also be involved in the neuroinflammatory response in PD [11]. The lymphocyte infiltration could then be a result of the dysfunctional blood-brain barrier (BBB) found in PD patients [33].

#### 2.1.1. Is There an External Trigger for Neuroinflammation in PD?

The neuroinflammatory response and microglia activation in PD could be triggered by an environmental initiation factor (e.g., toxins, neurotropic viral or bacterial infections, and pesticides) or by aggregated a-Syn proteins in Lewy bodies [34, 35], eliciting a self-perpetuating cycle of chronic inflammation. Apart from these triggering factors, the aged brain is characterized by increased sensitivity to glial cell stimulation, the so-called glial priming [36]. Primed microglia cells have upregulated expression of receptors that respond to deleterious stimuli (such as CD14, CD45, major histocompatibility complex (MCH) II, or Toll-like receptor TLR4) and mediate immune responses [36]. Nevertheless, the inflammatory response in PD could be a secondary effect of cellular damage and neuronal loss, as the injured dopaminergic neurons can release chemoattractants intensifying the pathological process [10]. There is no escaping the fact that while neuroinflammation may have some beneficial effects by removing byproducts of degeneration, uncontrolled inflammatory reactions may contribute to a self-perpetuating cycle that eventually leads to degeneration and death of dopaminergic neurons [37].

### 2.2. Intestinal Inflammation

Intestinal dysfunction, with constipation being the most prominent complaint, is a common nonmotor symptom in PD and may precede the onset of motor symptoms by decades [13]. The bidirectional communication between the central and enteric nervous system, the so-called “gut-brain axis,” has received growing attention due to its contribution to the pathogenesis of neurological disorders including PD [2, 38]. In fact, chronic intestinal inflammation may contribute to the pathogenesis of neurodegenerative disorders such as PD [12, 39]. Indeed, biopsies of colonic tissue retrieved from PD patients revealed an increased expression of proinflammatory cytokines, such as TNF-*α*, IL-1*β*, IL-6, and IFN-*γ*, as well as an increased activation of enteric glial cells [40]. Markers of intestinal inflammation (calprotectin) and permeability (alpha-1-antitrypsin and zonulin) [41] as well as IL-1*α*, IL-1*β*, CXCL8 (interleukin 8), and C-reactive protein (CRP) [42] were found to be elevated in stool samples of PD patients compared to healthy controls. Moreover, genetic studies support the connection between intestinal inflammation and PD. Genetic variants such as the leucine-rich repeat kinase 2 gene (LRRK2), which regulates inflammatory responses, are common in Crohn's disease, a chronic inflammatory intestinal disease, and familial as well as sporadic PD [43, 44]. Studies assessing genetic variants in the region of the nucleotide-binding oligomerization domain containing 2 (NOD2) gene, which are assumed to play a role in the pathogenesis of inflammatory bowel disease by modulating mucosal immunity [45], showed inconsistent results in PD [46, 47].

The intestine can modulate CNS activity via the vagus nerve or through the effects of the intestinal microbial community (microbiota), which releases signaling molecules into the systemic circulation and modulates the activity of immune cells [12, 48]. There is growing evidence that microbiota composition is altered in PD patients, and more specifically, it appears to be deficient in microbes with anti-inflammatory activities and enriched in microbes that have the potential to stimulate inflammation [12]. A lower abundance of bacteria with anti-inflammatory properties like short-chain fatty acid- (SCFA-) producing bacteria (i.e., Prevotella) is found in PD fecal samples [49, 50], which might contribute to increased gut permeability, probably through reduced production of mucin [51]. A small intestinal bacterial overgrowth (SIBO), a disorder with excessive bacterial growth in the small intestine, has also been reported in PD patients with a prevalence varying from 25% to 67% [52–55]. SIBO has been associated with gastrointestinal symptoms and worse motor scores (UPDRS III) [54, 55] while there is inconsistency regarding its effects on motor fluctuations [52, 54, 55]. SIBO might cause changes in intestinal permeability and contribute to an increase in bacterial translocation and consequently induction of an inflammatory response in the host [56].

Other inflammatory triggers such as a toxic substance (pesticides or pollutants) or a gastrointestinal infection could also possibly start the pathological process in the enteric nervous system, probably against a background of genetic vulnerability, causing mucosal inflammation and oxidative stress [57]. The following immune response could lead to changes in intestinal permeability, which is found to be increased in PD (leaky gut) [51], allowing microbial products such as endotoxins (LPS) and inflammatory mediators from the intestine to enter the systemic circulation [58]. The inflammatory response might then affect systemically the brain through a dysfunctional blood-brain barrier (BBB), which is found in PD patients [33]. Another possible mechanism is that inflammation in the gastrointestinal tract may initiate a-Syn accumulation in the enteric nervous system of PD patients [51, 59]. Aggregated and phosphorylated a-Syn, a hallmark of PD pathology, has been found at increased levels in the intestines of PD patients [60, 61], even in the early stages of PD [62, 63]. Yet, data concerning a-Syn in the enteric nervous system are controversial, as even phosphorylated forms (hypothesized to be a pathological form) are found in an age-dependent manner in otherwise healthy subjects [64, 65]. A-Syn deposition in neurons might begin in the enteric nervous system and then spread via the vagus nerve to the SN and other brain areas [2, 4]. Thus, synucleinopathy as well as inflammation would then spread throughout the brain affecting the dopaminergic neurons [12, 66]. This inflammatory cascade in PD could be further exacerbated by the aging-associated inflammation, which is also referred to as “inflammaging” [67].

## 3. Nonpharmacological Modulation of Inflammation in PD

### 3.1. Diet

The increasing evidence suggesting that the pathological process of PD may originate in the gut opens a potential new therapeutic window for the use of dietary strategies, which may influence the risk of developing PD or even modify the disease course [15]. Dietary interventions could influence the gut-brain axis by altering microbiota composition and modifying the production of biologically active microbial products or by direct interaction with immune cells or both [68]. Dietary components might also modulate the chronic inflammatory response that is associated with aging [69], the strongest risk factor for PD. Moreover, dietary interventions could potentially reduce gastrointestinal symptoms (constipation) and ameliorate levodopa uptake. Thus, the concept of nutraceuticals, which are compounds derived from natural food sources with scientific evidence for certain disease preventive and therapeutic effects when consumed as part of a varied diet on a regular basis and at optimal levels, is introduced in PD [70, 71].

Probiotics are specific microorganisms, which when administered in sufficient amounts can exert various health benefits. The most common are lactobacilli, enterococci, bifidobacteria, yeasts, and various mixtures of beneficial bacteria [72]. Probiotics are thought to suppress pathogenic bacterial growth, enhance the intestinal epithelial integrity by regulating intestinal tight junction protein expression, and contribute to the maintenance of the mucosal immune homeostasis [14, 73]. Probiotic strains can regulate defensin and antimicrobial protein secretion [74] and promote mucin production [75], thus enhancing the protective barrier mechanism in the intestine. Certain probiotic lipid antigens may also directly activate natural killer T cells [76]. Moreover, probiotics stimulate intestinal motility and reduce gastrointestinal dysfunction in elderly patients [77]. In PD, a study showed that a five-week intake of probiotics (fermented milk containing Lactobacillus casei Shirota) significantly improved stool consistency and bowel habits in PD patients [78]. Thus, probiotics could rebalance the PD-associated change in microbiota composition and thereby reduce gut leakiness, bacterial translocation, and the subsequent neuroinflammation. Prebiotics such as galactooligosaccharides (GOS) and fructooligosaccharides (FOS), which are nondigestible but fermentable oligosaccharides, stimulate the growth of beneficial gut microorganisms such as bifidobacteria and lactobacilli [79]. GOS and FOS are metabolized in the colon by bifidobacteria and create metabolic products such as SCFA, lactose, hydrogen, methane, and carbon dioxide, causing an acidic environment in the colon, which antagonizes the survival and proliferation of pathogenic bacteria [80]. SCFAs (acetate, lactate, propionate, and butyrate) contribute to the maintenance of intestinal epithelial integrity and regulate mucosal immune responses [81] by modulating the production of antimicrobial peptides and the secretion of proinflammatory mediators [82]. SCFA butyrate and propionate can also facilitate anti-inflammatory T regulatory cells generation [83, 84]. Moreover, prebiotic fibers have beneficial effects on intestine motility, as shown in a randomized controlled trial in PD [85]. The fecal concentrations of SCFA-producing bacteria are reduced in PD patients [49, 50], which could be rebalanced by the intake of prebiotic fibers. Indeed, a recent study showed that the intake of a digestion-resistant starch composition stimulates an increased abundance of beneficial bacteria (bifidobacteria) and results in an increase of SCFA butyrate in the stool of older adults [86]. The intake of this prebiotic could easily be applied in PD.

Dietary fats can influence intestinal inflammation and regulate mucosal immunity [87]. Polyunsaturated fatty acids (PUFAs), like the omega-3 fatty acid docosahexaenoic acid (DHA) found in fish oil, have anti-inflammatory effects [88]. Dietary *n*-3 PUFA inhibits the formation of proinflammatory prostaglandins and leukotrienes through the arachidonic acid pathway [89]. They also inhibit vascular adhesion and migration, angiogenesis, as well as adaptive immune responses by inhibiting T-cell proliferation and antigen presentation and by binding to nuclear receptors [89, 90]. Furthermore, long-chain *n*-3 PUFA affects cell membrane structure and inhibits activation of Toll-like receptor (TLR-4), which is important in mediating intestinal inflammation [91]. Indeed, two large prospective cohort studies showed that PUFA intake was associated with a reduced PD risk [92, 93]. The same was observed in ulcerative colitis, an inflammatory bowel disease [87]. Perez-Pardo et al. [94] showed that a diet containing fish oil (providing DHA) and uridine ameliorated intestinal barrier integrity and reduced gastrointestinal colonic inflammation as well as colonic a-Syn levels in mice PD models. It also protected from dopaminergic cell loss in the SN and improved motor deficits.

Phytochemicals are compounds found in plant-based foods with anti-inflammatory and antioxidant properties that are currently receiving attention in the prevention and treatment of different diseases. Caffeine, a nonselective antagonist of adenosine-2A receptors, has known anti-inflammatory properties. Caffeine is not only associated with a significantly lower risk of PD [95] (with the strongest protection at approximately 3  cups/day) [96] but also seems to have neuroprotective effects even after the onset of the neurodegenerative process, as shown in animal studies [97, 98]. Coffee results in an in vivo increase of anti-inflammatory bifidobacteria and a decrease of *Clostridium* spp. and *Escherichia coli* [99, 100], which may reduce intestinal inflammation. Caffeine also seems to attenuate neuroinflammation in animal models with LPS-induced and age-related inflammation by reducing microglia activation or via its ability to regulate glutamate release [101]. Caffeine may also increase the bioavailability of levodopa [102], while its effects on PD motor symptoms are inconsistent [103, 104]. Flavonoids (found in high concentrations in tea, cocoa, berry fruits, apples, red wine, and orange juice), a group of plant secondary metabolites known to have diverse biological activity in vivo [105], were associated with a 40% lower PD risk, although this finding was restricted to men [106]. Apart from their antioxidant properties, flavonoids have the potential to inhibit neuroinflammation through attenuation of microglial activation and associated cytokine release, as well as by suppressing the expression of oxidation-related enzymes (such as nitric oxide synthase (INOS), nitric oxide production, and NADPH oxidase activity) [105]. The regulation of these immune events appears to be mediated via intracellular signaling pathways, including the NF-*κ*B cascade and the mitogen-activated protein kinase (MAPK) pathway [105]. In fact, pure flavonoids (e.g., epigallocatechin-3-gallate) or enriched extracts can reduce the expression of proinflammatory cytokines (IL-6, TNF-*α*, IL-1*β*, and COX-2), downregulate inflammatory markers, and prevent neural damage [107]. Epigallocatechin-3-gallate (EGCG) is a compound (catechin) present in green tea, which crosses the BBB and has been shown to have neuroprotective properties in animal PD models [108, 109]. However, there was no association found between green tea consumption and PD risk [110]. Epigallocatechin-3-gallate is related to regulation of neuroinflammation and modulation of genes involved in cell survival by decreasing intracellular calcium levels and controlling nitric oxide production [111]. Experimental studies showed that administration of flavonoids protected dopamine neurons from oxidative damage and apoptosis [112, 113] and inhibited the formation of a-Syn fibrils [114].

Resveratrol, a type of natural phenol and a phytoalexin found in grapes, peanuts, berries, and pines, can inhibit the activation of microglia and the subsequent release of proinflammatory factors [115]. The anti-inflammatory action of resveratrol is attributed to the attenuation of the activation of MAPK and NF-*κ*B signaling pathways in microglia and the inhibition of NADPH oxidase with subsequent reduction of ROS generation [116]. Indeed, resveratrol reduced the inflammation-mediated apoptotic death of neuronal cells evoked by LPS-activated microglia in a microglia-neuronal coculture system [117]. Resveratrol was also shown to attenuate neurotoxicity in toxin-induced (MPTP-, 6-OHDA-, and LPS-) PD animal models [116, 118, 119]. Other phytochemicals with anti-inflammatory and antiapoptotic properties, which seem to be neuroprotective in animal models of PD, are sodium benzoate, the anti-inflammatory metabolite of cinnamon [120], curcumoinoids that constitute approximately 4% of turmeric [121], sulforaphane found in cruciferous vegetables such as broccoli and cabbage [122], ginkgo*-*biloba extract EGb 761, and the phytoestrogen genistein found in dietary soy and peanut products [71]. Clinical trials of the effects of these compounds on PD patients have not been conducted yet.

Micronutrients such as vitamins can also exert antioxidant and anti-inflammatory activities. Higher intake of vitamin B6 [123] and vitamin D [124] or moderate intake of vitamin E [125] might be associated with a lower PD risk. Vitamin B6 is reported to possess antioxidant [126, 127] and anti-inflammatory activities that seem to be independent of its homocysteine-lowering activity [128]. The protective effect of vitamin E on PD risk is plausible through reducing oxidative damage by neutralizing the effect of oxygen free radicals, as shown in in vivo studies [129]. 1,25-(OH)2D, the active form of vitamin D, inhibits the synthesis of iNOS [130] and its administration was shown to attenuate neurotoxicity in PD animal models [131].

Importantly, dietary strategies might also modulate the inflammatory mechanisms associated with cognitive decline, which is a common nonmotor symptom in PD [13]. In particular, increased consumption of total flavonoids was associated with a reduced rate of cognitive decline in adults aged 70 and over [132]. In animal studies, diets supplemented with berries (rich in flavonoids) improved the performance of aged rats in spatial working memory tasks [133]. Rabassa et al. [134] found that patients having a diet rich in polyphenols (>600 mg/d) had a lower risk of global cognitive decline but not of executive dysfunction. Moreover, there seems to be a potential correlation of moderate consumption of caffeine with a reduction in cognitive decline, as reviewed recently [135]. Nutritional interventions with *n*-3 PUFA might be beneficial in the earlier stages of cognitive impairment in older adults [136, 137]. Animal studies showed that probiotics such as *Lactobacillus* attenuated the LPS neuroinflammation-induced decline in performance in spatial learning tasks and this effect was accompanied by an increase of antioxidant enzymes and reduction of proinflammatory cytokines [138]. The effects of dietary interventions with prebiotics on cognitive outcomes in not yet demented adults have been quite inconsistent [139, 140]. Thus, it could be hypothesized that nutritional strategies might be of benefit in patients with PD-associated mild cognitive impairment or dementia, but such trials have not been conducted yet. The mechanisms, through which the different nutrients might affect age- and PD-related cognitive changes, need to be further elucidated, but it seems that their anti-inflammatory properties play a certain role [69].

The various published studies concerning diet and PD mainly focused on single nutrients rather than dietary patterns. However, the synergic and cumulative effects of different nutrients are important in a dietary pattern. The Mediterranean diet, characterized by high intake of fruits, vegetables, cereals, and olive oil, moderate intake of fish, dairy products, and wine, and lower consumption of meat, poultry, and saturated (animal) fats [141], is associated with a lower risk of PD or even prodromal PD [142–144]. The mechanisms, by which Mediterranean diet may attenuate inflammation and exert its positive effects on PD, may involve the antioxidant and anti-inflammatory activity of its components such as complex phenols, PUFA, vitamins C and E and carotenoid. Adherence to Mediterranean diet is associated with higher levels of adiponectin (an adipokine mostly secreted from adipose tissue with antidiabetic, antiobesity, and anti-inflammatory effects) and lower levels of TNF-*α*, high-sensitivity CRP, and IL-6 [145–147]. Furthermore, Mediterranean diet as a whole as well as its components have been associated with beneficial gut microbiota patterns [148, 149]. Thus, Mediterranean diet may exert a positive effect on intestinal inflammation and the gut-brain axis. Moreover, Peterrson et al. in a recent review [150] reported that adherence to Mediterranean diet is associated with improved cognitive function and decreased risk of cognitive impairment or dementia. This finding could have implications for patients with PD as well, particularly those at high risk of developing PDD.

### 3.2. Physical Activity and Exercise

Diet interventions in PD should be complemented by physical activity (PA) or regular and structured exercise, which are crucial for the maintenance of body and brain health. Their benefits in attenuating neuroinflammation have been reviewed previously [151, 152]; here, the main aspects will be reported briefly. Epidemiological studies show that a physically active lifestyle reduces the risk of PD by 33% to approximately 50% [153, 154]. PA may improve the absorption of levodopa, through increased mesenteric blood flow or accelerated gastric emptying [155]. Apart from its known beneficial effects on motor [156] and mental symptoms [157], it is proposed that PA may minimize brain diseases by modifying glia-mediated neuroinflammation [151]. Both acute and chronic exercises have immune-modifying properties, which lead to a whole-body anti-inflammatory effect [158].

Indeed, PA has anti-inflammatory effects in both the periphery and the central nervous system. PA may exert its anti-inflammatory properties in the periphery through several mechanisms: reduction of chronic oxidative stress [159, 160], reduction of LPS-stimulated secretion of proinflammatory cytokines and hsCRP [161, 162], and induction of the release of IL-6 into circulation from contracting muscle fibers [163]. Moreover, exercise reduces the expression of Toll-like receptors (TLR2 and TLR4) on monocytes [161, 164]. Furthermore, exercise can induce a release of (CD4^+^CD25^+^) T regulatory cells with an anti-inflammatory phenotype [165]. The anti-inflammatory effects of regular exercise may also be mediated by a reduction in visceral fat mass with a subsequent decreased release of adipokines from adipose tissue [158] and decreased number of proinflammatory monocytes (CD14^+^CD16^+^) [166] and macrophages infiltrating adipose tissue. In the CNS, the anti-inflammatory properties of PA may be due to direct effects on microglia by influencing their activity and proliferation [167]. Indeed, wheel running reduced the proportion of new microglia in the brains of adult mice [168, 169] and increased the proportion of microglia expressing insulin-like growth factor (IGF-1), a phenotype promoting neuroprotection, growth, and brain plasticity [167]. Cultured microglia from aged physically active rats had lower expression of IL-1*β* and TNF-*α* compared to microglia from sedentary aged rats [170]. Jang et al. [171] reported that endurance exercise suppressed a-Syn levels and reversed MPTP-induced neuroinflammation by hindering TLR2 downstream signaling cascades in the brain of MPTP-intoxicated mice. Lastly, PA could exert further beneficial effects by interfering with neuroinflammation through modulation of the kynurenine pathway [151].

PA has also beneficial effects with regard to mood and mental health through an increase of the neuronal release of serotonin and dopamine [152, 172]. Furthermore, it is associated with higher expression of brain-derived growth and neurotrophic factor (BDNF), vascular endothelial growth factor (VEGF), and glial cell line-derived neurotrophic factor (GDNF) in animal studies [173, 174], which enhance the survival and growth of neurons [162], delaying the neurodegenerative process. The synthesis of dopamine and trophic factors stimulated by PA inhibits neuroinflammation and apoptosis and promotes neuroplasticity [152]. Exercise-induced neurogenesis, synaptic strength, and angiogenesis may significantly contribute to the regeneration of neurons and thus restore normal motor functions [175, 176].

## 4. Conclusion

Neuroinflammation is increasingly recognized as an important pathophysiological feature of neurodegenerative diseases such as PD. Therefore, there is growing interest in developing therapeutic strategies targeting neuroinflammation in PD. Drugs to treat inflammation, such as NSAIDs, that have been shown to reduce the risk of PD in large epidemiological studies, have adverse effects, cross poorly the BBB, and rather halt the proinflammatory response than induce an “anti-inflammatory” response [11]. Thus, nonpharmacological interventions have received attention, in order to deal with neuroinflammation and potentially modify the course of the disease.

The data suggesting that neuroinflammation might start in the intestine open doors to food-based therapies, which may favorably modulate gut microbiota composition, enhance the intestinal epithelial integrity, and decrease the proinflammatory response [14]. Pre- and probiotics, diets rich in polyunsaturated fatty acids, phenols including flavonoids, and vitamins, such as the Mediterranean diet or a plant-based diet, may attenuate chronic inflammation and exert beneficial effects on PD symptoms [177] and even progression of the disease [178]. Of course, a better understanding of the gut-brain interactions and more specifically of the link between an altered gut microbiota composition, intestinal permeability, systemic and brain inflammation, and, eventually, neurodegeneration is required. Human studies should complement the in vitro and animal studies of dietary components and valuate the holistic effects of diet, taking into account the synergies and interactions of the different nutritional elements. However, the dosages of nutrients administered in animal studies vary largely or are even much higher compared to doses typically ingested by humans. Thus, these dosages can only be a rough estimate when designing human studies taking into account the metabolic alterations and bioavailability of nutrients in humans. Research in this field is challenging as there are difficulties in reliably assessing the dietary intake of individuals and even more the availability at the tissue level. Furthermore, it cannot be excluded that previous long-term exposure, i.e., previous dietary habits at a younger age, may be more relevant than the current diet [179]. This aspect might be particularly relevant considering the very long preclinical phase of PD. Furthermore, future studies should be of adequate duration (preferably longer than 1.5 years [180]) and possibly target patients with specific nutritional insufficiency. Further research is also required in the field of nutritional genomics, focusing on genetic variations that could eventually allow personalized nutrition [15].

In the context of a holistic multidimensional approach, physical activity has also emerged as a potent nonpharmacological intervention in the management of PD, which, combined with dietary interventions, might also interfere with PD pathophysiology. Nevertheless, further research is required in order to elucidate the exact neuroimmune mechanisms of exercise and define the favorable exercise intensity, as the amount of exercise in animal studies cannot be directly applied in patients with motor deficits such as PD patients. Both diet and physical activity target mediators of inflammation with various mechanisms and combined together as well as with dopaminergic treatment can increase their therapeutic benefits.

Indeed, engaging in physical activity and adhering to a healthy diet such as the Mediterranean diet can promote integrity and health of the aging brain [181]. Attention should also be drawn to the ways of educating and encouraging PD patients to adopt corresponding lifestyle modifications, in order to improve not only PD symptoms, but also general quality of life. The existing data, mostly from animal models of PD, provide strong scientific evidence to plan larger clinical trials with nonpharmacological and specifically dietary interventions in PD.
